# Experiences of Parental Presence in the Induction of Anesthesia in a Canadian Tertiary Pediatric Hospital: A Cross-Sectional Study

**DOI:** 10.7759/cureus.36246

**Published:** 2023-03-16

**Authors:** Gabriela Alcaraz Garcia-Tejedor, Matthew Le, Theophilus Tackey, Jessica Watkins, Monica Caldeira-Kulbakas, Clyde Matava

**Affiliations:** 1 Anesthesia and Pain Medicine, Hospital for Sick Children, Toronto, CAN; 2 Anesthesiology and Pain Medicine, University of Toronto, Toronto, CAN

**Keywords:** parental presence at induction of anesthesia, physician-parent interaction, preoperative anxiety, anxiety, pediatric anesthesia

## Abstract

Background

Parental presence at induction of anesthesia remains controversial and has been reported to provide mixed results. As such, parental presence at induction of anesthesia is not practiced routinely everywhere. There are currently limited data describing the practice of parental presence at induction of anesthesia or the experiences and perceptions of parents in Canada.

Objectives

We sought to investigate (1) the frequency of parental presence at induction of anesthesia and (2) the experiences and perceptions of parents accompanying their child into the operating room compared to those who did not at a tertiary Canadian pediatric hospital.

Methods

Institutional quality improvement approval was obtained. This study was a cross-sectional survey. Parents waiting in the parent surgical waiting room during the procedure were invited to complete a web-based survey. Consent was implied via completing the survey. The cross-sectional survey elicited the prevalence of parental presence during induction of anesthesia as well as their experience and perceptions. We also investigated the parents’ preferences for preoperative education.

Results

Of the 448 parents approached, 403 completed the survey between May and June 2017. Sixty-eight (16.9% [13.4-20.9]) parents accompanied their child into the operating room (parental presence at induction of anesthesia), while 335/403 (83.1% [79.1-86.7]) did not (no-parental presence at induction of anesthesia). Reasons for not accompanying their child into the operating room included “not being aware they could” (158/335, 47.2% [41.9-52.5]), “I didn’t think my child needed me” (107/335, 31.9% [27.2-37.1]), “my child was coping well” (46/335, 13.4% [10.5-17.8]), and “I was anxious” (47/335, 14.0% [10.7-18.2]). Most of the parents in the parental presence at induction of anesthesia cohort (66/67, 98.5% [95.6-101.2]) reported that they believed their child benefited/would have benefited from their presence during induction of anesthesia compared to those in the no-parental presence at induction of anesthesia cohort (137/335, 40.9% [35.8-46.2]), P < 0.001. Overall, 51/335 (14.7%) parents in the no-parental presence at induction of anesthesia cohort and 3/67 (4.5%) of those in the parental presence at induction of anesthesia cohort felt that offering parental presence at induction of anesthesia should depend on factors including child’s age as well as the level of coping and anxiety. More patients in the no-parental presence at induction of anesthesia cohort felt that parental presence at induction of anesthesia should also depend on the child's age and whether the child was coping. Parents felt that face-to-face discussions with clinicians are most effective for discussing future parental presence at induction of anesthesia.

Conclusions

We have shown that most parents at our institution do not undergo parental presence at induction of anesthesia and are for the most part comfortable with their child going unaccompanied into the operating room. Administrators and clinicians seeking to implement parental presence policies should consider navigating parental presence at induction of anesthesia with evidence-based approaches tailored to each parent and their child.

## Introduction

The preoperative experience can be fraught with anxiety and uncertainty for parents of pediatric surgical patients. Techniques available to the anesthesiologist to prevent and manage preoperative anxiety in children include premedication, distraction techniques (videos, games, bubbles, clowns, virtual, and immersive reality), child life specialists, and parental presence at induction of anesthesia (PPIA) [1-8]. The benefits of PPIA remain controversial, and PPIA is not a part of routine practice everywhere [4-12]. In the United States, 58% of anesthesiologists have reported allowing parental presence in less than 5% of their cases [13]. In contrast, in Great Britain, most respondents (84%) allowed parental presence in more than 75% of their cases [13]. Parents experiencing PPIA have reported it as traumatizing or distressing to witness, with variable feelings in parents who decided not to attend induction (no-PPIA) and mixed feelings in the interactions with the care teams along with positive feelings [9,14]. Currently, there are no data describing the prevalence of PPIA in Canada as well as the experiences and perceptions of parents who were present or not present during the induction of anesthesia of their child.

The objectives of our study were to investigate the prevalence of parental presence during induction of anesthesia and explore parents' experience and perceptions of PPIA at a Canadian pediatric tertiary-care academic hospital. We also compared parents’ experiences and preferences for preoperative education on PPIA. The results of this survey will help inform policies guiding PPIA at our institution and may be relevant to other institutions.

## Materials and methods

Ethics and setting

The study received approval from the Hospital for Sick Children's Risk and Management Committee as a quality improvement initiative. The survey was administered on an iPad™ to parents after their child was taken to the operating room for elective surgery at a Canadian pediatric tertiary-care academic hospital [15]. Parents were approached by the research assistant and were invited to participate in the study. Completion of the survey implied consent.

Participants

The survey was presented to a convenience sample of parents and parents who were waiting in the surgical waiting room during the procedure. Participants were assured that their participation was voluntary, and the information they provided remained confidential with the results being reported aggregate. Completion of the survey was taken as consent. Parents who did not speak English and those who did not consent to complete the survey were excluded. 

Survey design

Following a literature review of other similar surveys, we designed the survey and pretested it on volunteer parents [11,13,16-19]. No significant changes were made after feedback from the parents. Following pretesting among the authors and a pilot among eight parents, the final survey tool was loaded onto an iPad™ for administering to parents between May and June 2017. The responses from the testing and the pilot phases were not included in the analysis.

The final survey instrument consisted of five demographic questions for all participants, which then branches based on whether the parent/caregiver was in the PPIA cohort or the no-PPIA cohort. Parents in the PPIA cohort responded to six questions specific to their PPIA experience, while parents who did not accompany their child responded to six questions tailored to their no-PPIA experience. The survey concluded with three questions common to both cohorts.

Sample size and sampling

Based on a target population of 14,000 anesthetics each year for surgery alone at our institution, we required a minimum of 384 respondents to achieve a 95% confidence level with a 5% error margin [20-22]. To minimize sampling bias, we used a stratified sampling approach. We recruited 15 patients each day from the parent surgical waiting area. The research assistant administering the survey had no knowledge of whether parents were in the PPIA or no-PPIA cohort. The patient waiting area is located on a different part of the operating room floor, across several doors and corridors, preventing the research assistant from seeing which parents had been in the operating room. When each parent entered the waiting area, the assistant tossed a coin with heads denoting that they can be approached to participate in the survey. Each parent who had heads in the coin toss was approached until 15 patients were recruited each day. We also recruited 10 parents in the morning, between 8.30 am and noon when more operating rooms were open, and five parents in the afternoon, between 1 pm and 3 pm. This allowed us to sample from a wide pool of parents. Each day, 40-50 parents entered the parent waiting area, and almost half of them were approached for the study. The data was collected over a four-week period.

Statistical analysis

Descriptive statistics were used to summarize the results. Categorical variables are presented as frequency and proportion (95% confidence intervals). Pearson's Chi-square test was used to assess the difference in perceptions between the two cohorts on three questions asking how they felt their child benefited/would have benefited from their presence, whether PPIA should be offered to all parents, and if they would want to be present in the operating room in the future. The statistical significances were defined as P-value ≤ 0.05 with a two-tailed test. The survey is reported according to the Checklist for Reporting Results of Internet E-Surveys (CHERRIES) [23]. Statistical analyses were performed using SAS software, version 9.4 (SAS Institute, Cary, North Carolina).

## Results

Demographics

A total of 448 parents/caregivers were approached, with 46 declining and 402 participating in the survey. The demographics of parents participating in the study are presented in Table 1. One respondent in the PPIA cohort did not complete the remaining survey questions. Of those in the PPIA cohort, 68/68 (100% [94.7-100]) were parents of the child compared to 300/335 (89.6% [85.8-92.6]) in the no-PPIA cohort. Those who did not accompany the child were older parents, including guardians, and older children (Table 1).

**Table 1:** Demographics of respondents *PPIA: Parental presence at induction of anesthesia. **No-PPIA: No-parental presence at induction of anesthesia.

Item	PPIA* (n = 68)	% [95% CI]	no-PPIA** (n = 335)	% [95% CI]
*What is your relationship to the child you brought to the hospital today?*				
Parent	68	100 [94.7-100]	300	89.6 [85.7-92.6]
Other family members	0 [0.00]	0 [0-5.3]	31	9.3 [6.4-12.9]
Legal guardian	0 [0.00]	0 [0-5.3]	4	1.2 [0.3-3.0]
*Age range*				
<25 years old	11	16.2 [8.4-27.1]	35	10.5 [7.4-14.2]
25-35 years old	34	50 [37.6-62.4]	136	40.6 [35.3-46.1]
36-45 years old	19	27.9 [17.7-40.1]	95	28.4 [23.6-33.5]
>45 years old	4	5.9 [1.6-14.4]	69	20.1 [16.4-25.3]
*Highest level of schooling completed*				
Primary school	0	0 [0-5.3]	2	0.6 [0.07-2.1]
High school	13	19.1 [10.6-30.5]	62	18.5 [14.5-23.1]
College or diploma	20	29.4 [19.0-41.7]	93	27.8 [23.0-32.9]
University degree	28	41.2 [29.4-53.8]	113 [33.73]	33.7 [28.7-39.1]
Postgraduate degree	6	8.8 [3.3-18.2]	65 [19.40]	19.4 [15.3-24.1]
Other	1	1.5 [0.04-7.9]	0 [0.00]	0 [0-1.1]
*How many anesthetics have you had personally?*				
0	30	44.1 [32.1-56.7]	133	39.7 [34.4-45.2]
1	13	19.1 [10.6-30.5]	72	21.5 [17.5-26.3]
2	15	22.1 [12.9-33.8]	81	24.2 [19.7-29.1]
3	3	4.4 [0.9-12.4]	26	7.8 [5.1-11.2]
4	3	4.4 [0.9-12.4]	11	3.3 [1.7-5.8]
>4	4	5.9 [1.6-14.4]	12	3.6 [1.9-6.2]
*How old is the patient you accompanied today?*				
<2 years old	23 [33.82]	33.8 [22.8-46.3]	132	39.4 [34.1-44.9]
2-5 years old	31	45.6 [33.5-58.1]	85	25.4 [20.8-30.4]
6-12 years old	13	19.1 [10.6-30.5]	71	21.2 [16.4-26.0]
>12 years old	1	1.5 [.04-7.9]	47	14.0 [10.5-18.2]

Experiences in the parental presence at induction of anesthesia cohort

Sixty-eight (16.9%, 13.4-20.9) parents accompanied their child into the operating room, PPIA cohort. The most common reason given by parents for accompanying the child into the room was “I wanted to” 50/67 (74.6% [63.1-83.5]) (Table 2).

**Table 2:** Experiences and perceptions of parents/guardians in the PPIA cohort (n = 67) *“Precautionary” was mentioned one time: 1.5 [0.3-8]; “Better for the child” was mentioned two times: 3 [0.8-10.3]; “Not sure why” was mentioned one time: 1.5 [0.3-8]. PPIA: Parental presence at induction of anesthesia; OR: Operating room.

Item	n	% [95% CI]
Reason for accompanying the child		
I wanted to	50	74.6 [63.1-83.5]
My child wanted me to	43	64.2 [52.2-74.6]
The nurse suggested I should	0	-
The anesthesiologist suggested I should	1	1.5 [0.3-8]
The surgeon suggested I should	1	1.5 [0.3-8]
The child life specialist suggested I should	0	-
Others*	4	6 [2.4-14.4]
How did you prepare prior to entering the OR?		
Researched the hospital website	7	10.5 [5.2-20.0]
Researched different websites	4	6 [2.4-14.4]
Referred to the pamphlets that were provided	9	13.4 [7.2-23.6]
Referred to the videos that were provided	2	3 [0.8-10.3]
Prepared by the nurse	32	47.8 [36.3-59.5]
Prepared by the anesthesiologist	42	62.7 [50.7-73.3]
Prepared by the surgeon	11	16.4 [9.4-27.1]
Prepared by the child life specialist	1	1.5 [0.3-8]
Previous experience	6	9 [4.2-18.2]
How informed were you prior to entering the OR?		
Not at all informed	0	-
Somewhat not informed	0	-
Neutral	0	-
Somewhat informed	6	9 [4.2-18.2]
Very well informed	61	91.0 [81.8-95.8]
How prepared were you prior to entering the OR?		
Not prepared	0	-
Somewhat not prepared	0	-
Neutral	0	-
Somewhat prepared	4	6 [2.4-14.4]
Well prepared	63	94 [85.6-97.7]

Most parents (42/67, 62.7% [50.7-73.3]) were prepared by the anesthesiologist prior to entering the operating room (Table 2). Most parents who accompanied their child into the operating room felt they were “very well informed prior to entering the OR” (61/67, 91% [81.8-95.8]) and “very well prepared” (63/67, 94% [85.6-97.7]).

Experience of parents in the no-parental presence at induction cohort

A total of 335 out of 403 (83.1% [79.1-86.7]) parents did not accompany their children into the operating room in the no-PPIA cohort. Reasons for not accompanying their child into the operating room included “not being aware they could” (158/335, 47.2% [41.9-52.5]), “I didn’t think my child needed me” (107/335, 31.9% [27.2-37.1]), “my child was coping well” (46/335, 13.4% [10.5-17.8]), and “I was anxious” (47/335, 14.0% [10.7-18.2]) (Table 3).

**Table 3:** Perceptions and experiences from caregivers in the no-PPIA cohort No-PPIA: No-parental presence at induction of anesthesia.

Item	n	% [95% CI]
*Reason for not accompanying your child*		
I was anxious/nervous	47	14.0 [10.7-18.2]
I did not feel I would have been helpful	45	13.4 [10.2-17.5]
I did not think my child needed me	107	31.9 [27.2-37.1]
I was told I couldn’t by the nurse	3	0.9 [0.3-2.6]
I was told I couldn’t by the anesthesiologist	3	0.9 [0.3-2.6]
I was told I couldn’t by the surgeon	0	-
My child appeared to be coping well	46	13.7 [10.5-17.8]
I was not aware I could	158	47.2 [41.9-52.5]
Others	32	9.6 [6.9-13.2]
*How do you feel about not being present while your child was put to sleep?*		
Negative	6	1.8 [0.8-3.9]
Positive	53	15.8 [12.3-20.1]
Bothered	13	3.9 [2.3-6.5]
Not bothered	47	14.0 [10.7-18.2]
Uncomfortable	21	6.3 [4.1-9.4]
Comfortable	141	42.1 [36.9-47.4]
I am sure it went well	61	18.2 [14.5-22.7]
Anxious	35	10.5 [7.6-14.2]
*Do you feel you would have benefited from being present while your child was put to sleep?*		
Yes	163	48.7 [43.4-54]
No	108	32.2 [27.5-37.4]
Maybe	64	19.1 [15.3-23.7]

However, most parents were comfortable/felt positive about not accompanying their child into the OR (Table 3); 170/335 (51%) of parents who did not accompany their child into the operating room stated that they would have liked to, and 163/335 (48.7% [43.4-54]) felt they would have personally benefited from being presented in the OR.

Comparisons of experiences and perceptions between the PPIA and no-PPIA cohorts

More parents in the PPIA cohort (66/67, 98.5% [95.6-101.2]) reported that they believed their child benefited/would have benefited from their presence during induction of anesthesia compared to those in the no-PPIA cohort (137/335, 40.9% [35.8-46.2]), P < 0.001. Most parents in the PPIA cohort (42/67, 62.7% [50.0-74.2]) compared to the no-PPIA cohort (149/335, 44.5% [39.1-50.0]) felt that PPIA should be always offered to all parents, P < 0.001. Overall, 51/335 (15.2% [11.6-20.0]) of parents in the no-PPIA cohort and 3/67 (4.5% [1.0-12.5]) of those in the PPIA cohort felt that offering PPIA should depend on factors including child’s age and the level of coping and anxiety. Significantly, more parents in the PPIA cohort (48/67, 71.6% [59.3-82.0]) would absolutely want to be present during future inductions of their child compared to the no-PPIA cohort (94/335, 28.2% [23.6-33.5]) (P < 0.001). More patients in the no-PPIA cohort (98/335; 28.2% [23.6-33.5]) compared to the PPIA cohort (5/335; 5% [2.5-16.6]) felt that their presence should depend on the age of the child, type of surgery, and whether their child needed them or was coping (Table 4).

**Table 4:** Comparison of caregiver experiences and perceptions between the two cohorts, PPIA versus no-PPIA *PPIA: Parental presence at induction of anesthesia. **No-PPIA: No-parental presence at induction of anesthesia. ^†^Respondents selecting it depends on entered free text with the themes “Age of child,” n = 46 [12.4]; “Type of surgery,” n = 14; “Physician discretion,” n = 3; “Whether my child needed me,” n = 37. ^††^Respondents selecting it depends on entered free text with the themes “Age of child,” n = 50 [12.4]; “Type of surgery,” n = 33; “Physician discretion,” n = 3; “Whether my child needed me,” n = 13.

Item	PPIA* (n = 67)		no-PPIA** (n = 335)		P-value
	n	% [95% CI]	n	% [95% CI]	
Do you feel your child benefited/would have benefited from your presence?					
Yes	66	98.5 [95.6-101.2]	137	40.9 [35.8-46.2]	<0.001
No	0	-	169	50.5 [44.1-56.0]	
Maybe	1	1.5 [1.4-4.4]	29	8.7 [5.9-12.2]	
Should caregiver presence be offered to all caregivers?					
Not at all	0	-	0	-	<0.001
Only under rare circumstances	0	-	14	4.2 [2.3-6.9]	
Neutral	0	-	8	2.4 [1.0-4.7]	
Most of the time	22	32.8 [21.9-45.4]	149	44.5 [39.1-50.0]	
Always	42	62.7 [50.0-74.2]	125	37.3 [32.1-42.7]	
It depends^†^	3	4.5 [1.0-12.5]	51	15.2 [11.6-20.0]	
In the future, would you want to be present in the OR while one of your children went to sleep?					
Definitely not	0	-	1	0.3 [0-1.4]	<0.001
Most likely not	0	-	21	6.2 [3.9-9.4]	
Neutral	1	1.5 [0.0-8.0]	30	9.0 [6.1-12.5]	
Most likely	13	19.4 [10.8-30.9]	94	28.1 [23.3-33.2]	
Absolutely	48	71.6 [59.3-82.0]	94	28.1 [23.3-33.2]	
It depends^††^	5	7.5 [2.5-16.6]	95	28.2 [23.6-33.5]	

Most parents in both cohorts, PPIA (50/67, 74.6%[63.1-83.5]) and no-PPIA (274/335, 81.8% [77.3-85.6]), reported that they would “talk calmly to my child” to support their child during induction of anesthesia (Table 5).

**Table 5:** Comparison of support for patients suggested to parents by caregivers in the PPIA group versus those considered by parents in the no-PPIA group *PPIA: Parental presence at induction of anesthesia. **no-PPIA: No-parental presence at induction of anesthesia. ^†^“Hold mask to administer anesthesia” was mentioned six times: 9 [4.2-18.2]. ^††^“Give pacifier” was mentioned one time: 0.3 [0.1-1.7]. “Pray for the child” was mentioned two times: 0.6 [0.2-2.2]. “Give the child money” was mentioned one time: 0.3 [0.1-1.7]. “Give the child a stuffed animal” was mentioned one time: 0.3 [0.1-1.7].

Item	PPIA* (n = 67)		no-PPIA** (n = 335)	
What did you do/would do to support your child during the induction of anesthesia?	n	% [95% CI]	n	% [95% CI]
Caress	10	14.9 [8.3-25.4]	151	45.1 [39.8-50.4]
Distract	42	62.7 [50.7-73.3]	192	57.3 [52-62.5]
Observe	38	56.7 [44.8-67.9]	132	39.4 [34.3-44.7]
Communication	2	3 [0.8-10.3]	53	15.8 [12.3-20.1]
Cuddle	7	10.5 [5.2-20.0]	121	36.1 [31.2-41.4]
Restrain	22	32.8 [22.8-44.8]	90	26.9 [22.4-31.9]
Sing	1	1.5 [0.3-8]	54	16.1 [12.6-20.4]
Talk calmly to my child	50	74.6 [63.1-83.5]	274	81.8 [77.3-85.6]
Other	6^†^	9 [4.2-18.2]	5^††^	1.5 [0.6-3.7]

Table 6 shows the preferences of parents on receiving information related to caregiver presence during the induction of anesthesia. A minority of all parents would review online resources to prepare for future PPIA. Most parents (376/402, 93.5% [91.1-96]) reported that face-to-face discussions would be the most effective for them when preparing for caregiver presence during the induction of anesthesia. Furthermore, parents would like discussions to include information about whether or not they would be allowed in the operating room (Table 6).

**Table 6:** Perceptions of the available resources on PPIA and preoperative preparation In the free-text comments, “Previous experience” was mentioned six times: 9 [4.2-18.2]. PPIA: Parental presence at induction of anesthesia; OR: Operating room.

Item	n	% [95% CI]
Websites visited for information on PPIA		
SickKids website [aboutkidshealth.ca]	60	14.9 [11.8-18.8]
Different hospital website	15	3.7 [2.3-6.1]
Not applicable	332	82.6 [78.6-86]
What is the most effective way to prepare parents for PPIA?		
Website	37	9.2 [6.4-12.1]
Pamphlet	37	9.2 [6.4-12.1]
Video	14	3.5 [1.7-5.3]
Face to face	376	93.5 [91.1-96]
Regarding PPIA, what kind of information should be provided to parents prior to their child’s surgery?		
Reasons why they are allowed in the OR	257 [63.9]	63.9 [59.2-68.6]
Reasons why they are not allowed in the OR	239 [59.5]	59.5 [54.7-64.3]
Who decided if they are allowed in the OR?	224 [55.7]	55.7 [50.9-60.6]
What happens as their child goes off to sleep?	302 [75.1]	75.1 [70.8-79.4]
How they can help?	315 [78.4]	78.4 [74.3-82.3]
How long they can stay in the OR?	249 [61.9]	61.9 [57.2-66.7]

Table 7 shows the perceptions of the information that parents had accessed online prior to their PPIA.

**Table 7:** Perceptions on internet-based information for PPIA PPIA: Parental presence at induction of anesthesia.

Item	n = 67	% [95% CI]
*For internet-based information, please indicate your perceptions of the information you found*		
Easy to find	30 [7.5]	44.8 [32.6-57.4]
Difficult to find	1 [0.2]	1.5 [0.04-8.0]
Helpful in preparing you for today	38 [9.5]	56.7 [44.0-68.8]
Not helpful in preparing you for today	1 [0.2]	1.5 [0.04-8.0]
Complete	27 [6.7]	40.3 [28.5-53.0]
Incomplete	1 [0.2]	1.5 [0.04-8.0]
Easy to understand	34 [8.5]	50.8 [38.2-63.2]
Difficult to understand	1 [0.2]	1.5 [0.04-8.0]
Useful	51 [12.7]	76.1 [64.1-85.7]
Not useful	1 [0.2]	1.5 [0.04-8.0]

## Discussion

Our study shows that most of the parents at our institution do not accompany their children into the operating room and are comfortable and accepting of this. Parents with younger children and previous experience with PPIA more often accompanied their children into the operating room. Overall, parents preferred a face-to-face discussion regarding the advantages and disadvantages of PPIA and would consider their child's age and ability to cope as factors influencing PPIA.

The finding that most parents attending our institution do not accompany their children into the operating is not surprising. A historical survey from 1996 administered to members of the Society of Pediatric Anesthesiologists reported a low prevalence of PPIA across the United States. About 58% of anesthesiologists in that study reported allowing parental presence in less than 5% of their cases compared to more than 84% of respondents in Great Britain who allowed parental presence in more than 75% of their cases [13]. While no updated data have been reported from the United State or Great Britain, our study shows a higher rate of PPIA compared to the United States. More importantly, our study attempts to shed light on the reasons and perspectives of both PPIA and no-PPIA cohorts. An interesting reason for no-PPIA among parents in our study population is that they stated that “their child didn’t need them” or “was coping well.” This suggests that a good proportion of children attending our institution can cope well and may be well prepared for their procedure and anesthetic through mechanisms not assessed by this current survey. Parents in the no-PPIA cohort also reported that the child’s age and ability to cope as well as the anesthesiologist’s assessment of the child were factors in determining whether they should be present during the induction of anesthesia or not. While our study did not assess the child’s or parent’s anxiety levels, anesthesiologists in pediatric settings have been shown to be better than mothers and trainees in predicting the anxiety of children during induction of anesthesia [24].

While most parents in the no-PPIA group wished they could be present, they reported their experience as positive and were comfortable with not being present for PPIA. One in 10 parents in the no-PPIA cohort reported their own anxiety as the reason for not accompanying their child to the operation room. Other studies have reported some parents experiencing PPIA describing it as traumatizing or distressing to witness, feeling “your world is not my world” and have also experienced mixed feelings in the interactions with the care teams [9,10,14]. Clinicians and administrators may need to consider the unintended consequences arising from the policy that “mandates” PPIA and may not offer the appropriate preparation and support for PPIA. In addition, some parents or children may have no desire for PPIA as their child may be coping well or exercising their autonomy in medical-decision making with the parent’s support [25-28]. As a result, clinicians and administrators will need to navigate and offer PPIA in a manner that acknowledges children who do not desire or require PPIA and parents who may want to give their child independence in the preoperative setting and during induction of anesthesia.

In our study, parents who accompanied their child into the PPIA were of younger age, had previous experience with PPIA, and reported the desire to do so in the future. Parents in the PPIA cohort also reported their presence as beneficial to the child. While our study did not assess either the parent's or the child's anxiety during anesthesia induction, studies have shown that in most cases, parental presence does not appear to affect either the parent's or the child's anxiety - premedicating children, toys, videos, and internet-based cognitive therapy; the presence of child life specialists or clowns are viable alternatives for reducing the child's anxiety [1,5,6,29-35]. Several parents in the no-PPIA cohort believed that PPIA would have been beneficial for themselves and suggested that it be offered to all parents always, and they were less likely to consider the child’s age or coping as a factor informing PPIA. This may be explained by the fact that parents in the PPIA group were of younger children who had not yet developed independence or autonomy with the healthcare system [25].

Our study also reports that parents prefer face-to-face discussions as the most effective way for discussing the potential for PPIA. Parents also preferred a discussion on the reasons why they may or may not accompany their child to the operating room. In contrast, a small proportion of parents felt online resources were effective for future PPIA encounters. However, those who had used online resources found them useful for this purpose. This finding is useful for administrators and clinicians who may be developing training materials for PPIA and will need to ensure these meet a variety of parental preferences for online or in-person information [22,31-45].

There are several limitations to the study. We restricted our study to the English language, which may have reduced representation from parents among minority groups and immigrants. We hope to translate future surveys into languages spoken by our patient populations. Further, as this was out of the scope of this study, we did not collect data on the process used for offering PPIA to parents. The use of premedication and other non-pharmacological agents may have influenced the offering of PPIA. The COVID-19 pandemic may have changed attitudes and practices around parental presence during the induction of anesthesia. Our study occurred before COVID and may not have captured these changes as a result of temporary restrictions imposed on parental presence in critical care areas [46,47]. Finally, our study did not assess patient perspectives on PPIA or no-PPIA, nor did we assess the anxiety of the parent or child during the induction of anesthesia [48-51]. These data are important in acquiring information to help clinicians and administrators navigate the role of PPIA. Nevertheless, our study had an adequate sample size to generate responses that are statistically representative of our English language population.

## Conclusions

We have shown that most parents at our institution do not undergo PPIA and are for the most part comfortable and accepting of this. Further studies seeking the child’s perspective on PPIA are warranted. Administrators and clinicians seeking to implement parental presence policies should consider navigating this area with evidence-based approaches tailored to each parent and their child.

## References

[1] Manyande A, Cyna AM, Yip P, Chooi C, Middleton P (2015). Non-pharmacological interventions for assisting the induction of anaesthesia in children. Cochrane Database Syst Rev.

[2] Marquez JL, Wang E, Rodriguez ST (2020). A retrospective cohort study of predictors and interventions that influence cooperation with mask induction in children. Paediatr Anaesth.

[3] Henderson MA, Baines DB, Overton JH (1993). Parental attitudes to presence at induction of paediatric anaesthesia. Anaesth Intensive Care.

[4] Wright KD, Stewart SH, Finley GA (2010). When are parents helpful? A randomized clinical trial of the efficacy of parental presence for pediatric anesthesia. Can J Anaesth.

[5] Chundamala J, Wright JG, Kemp SM (2009). An evidence-based review of parental presence during anesthesia induction and parent/child anxiety. Can J Anaesth.

[6] Golan G, Tighe P, Dobija N, Perel A, Keidan I (2009). Clowns for the prevention of preoperative anxiety in children: a randomized controlled trial. Paediatr Anaesth.

[7] Shih MC, Elvis PR, Nguyen SA, Brennan E, Clemmens CS (2023). Parental presence at induction of anesthesia to reduce anxiety: a systematic research and meta-analysis. J Perianesth Nurs.

[8] Mayo DG, Carretero PS, Martin LG, Calderón JA, Oliveros FH, Rojo MG (2022). Parental presence during induction of anesthesia improves compliance of the child and reduces emergence delirium. Eur J Pediatr Surg.

[9] Waseem H, Mazzamurro RS, Fisher AH, Bhowmik S, Zaman RA, Andrew A, Bauer DF (2018). Parental satisfaction with being present in the operating room during the induction of anesthesia prior to pediatric neurosurgical intervention: a qualitative analysis. J Neurosurg Pediatr.

[10] Aljohani DM (2022). Views, experiences, and challenges of anesthetists and anesthesia technologists on parental presence during induction of anesthesia in children: a mixed method study. Global Journal on Quality and Safety in Healthcare.

[11] Andersson L, Almerud Österberg S, Årestedt K, Johansson P (2022). Nurse anesthetist attitudes towards parental presence during anesthesia induction-a nationwide survey. J Adv Nurs.

[12] Velayos M, Estefanía K, Álvarez M (2021). Healthcare staff as promoters of parental presence at anesthetic induction: net promoter score survey. World J Clin Pediatr.

[13] Kain ZN, Ferris CA, Mayes LC, Rimar S (1996). Parental presence during induction of anaesthesia: practice differences between the United States and Great Britain. Paediatr Anaesth.

[14] Le Roux JJ, Redelinghuys C (2022). Attitudes and perceptions of caregivers regarding their presence at induction of anesthesia. Paediatr Anaesth.

[15] Matava CT, Williams RJ, Simpao AF (2022). An open-source toolkit to assist authors and collaborators during manuscript preparation: AuthorAndCollaborator toolkit. Can J Anaesth.

[16] Leelanukrom R, Somboonviboon W, Sriprachittichai P (2002). Parental presence during induction of anesthesia in children: a study on parental attitudes and children's cooperation. J Med Assoc Thai.

[17] Bösenberg AT, Williams GD, Reddy D (1996). Attitudes towards parental presence at induction of anaesthesia. S Afr Med J.

[18] Kain ZN, Fernandes LA, Touloukian RJ (1996). Parental presence during induction of anesthesia: the surgeon's perspective. Eur J Pediatr Surg.

[19] Zelikovsky N (1996). Parental participation during induction stage of children's anesthetic procedures in Israel. Semin Perioper Nurs.

[20] Story DA, Tait AR (2019). Survey research. Anesthesiology.

[21] Passmore C, Dobbie AE, Parchman M, Tysinger J (2002). Guidelines for constructing a survey. Fam Med.

[22] Pehora C, Gajaria N, Stoute M, Fracassa S, Serebale-O'Sullivan R, Matava CT (2015). Are parents getting it right? A survey of parents' internet use for children's health care information. Interact J Med Res.

[23] Eysenbach G (2004). Improving the quality of web surveys: the checklist for reporting results of internet e-surveys (CHERRIES). J Med Internet Res.

[24] MacLaren JE, Thompson C, Weinberg M, Fortier MA, Morrison DE, Perret D, Kain ZN (2009). Prediction of preoperative anxiety in children: who is most accurate?. Anesth Analg.

[25] Coughlin KW (2018). Medical decision-making in paediatrics: infancy to adolescence. Paediatr Child Health.

[26] Alderson P, Cohen M, Davies B (2022). The involvement and autonomy of young children undergoing elective paediatric cardiac surgery: a qualitative study. J Cardiothorac Surg.

[27] Ranganathan K, Luby AO, Haase M (2020). Decision making in pediatric plastic surgery: autonomy and shared approaches. J Craniofac Surg.

[28] Stavleu DC, Peter de Winter J, Veenstra X, van Stralen KJ, De Coninck D, Matthijs K, Toelen J (2022). Parental opinions on medical decision-making in adolescence: a case-based survey. J Dev Behav Pediatr.

[29] Lopes-Júnior LC, Bomfim E, Olson K (2020). Effectiveness of hospital clowns for symptom management in paediatrics: systematic review of randomised and non-randomised controlled trials. BMJ.

[30] Vagnoli L, Caprilli S, Messeri A (2010). Parental presence, clowns or sedative premedication to treat preoperative anxiety in children: what could be the most promising option?. Paediatr Anaesth.

[31] Gold JI, Annick ET, Lane AS, Ho K, Marty RT, Espinoza JC (2021). "Doc McStuffins: Doctor for a Day" virtual reality (DocVR) for pediatric preoperative anxiety and satisfaction: pediatric medical technology feasibility study. J Med Internet Res.

[32] Ridout B, Kelson J, Campbell A, Steinbeck K (2021). Effectiveness of virtual reality interventions for adolescent patients in hospital settings: systematic review. J Med Internet Res.

[33] Olbrecht VA, O'Conor KT, Williams SE (2021). Guided relaxation-based virtual reality for acute postoperative pain and anxiety in a pediatric population: pilot observational study. J Med Internet Res.

[34] Pan S, Rong LQ (2021). Mobile applications in clinical and perioperative care for anesthesia: narrative review. J Med Internet Res.

[35] Shahnavaz S, Hedman-Lagerlöf E, Hasselblad T, Reuterskiöld L, Kaldo V, Dahllöf G (2018). Internet-based cognitive behavioral therapy for children and adolescents with dental anxiety: open trial. J Med Internet Res.

[36] O'Sullivan B, Alam F, Matava C (2018). Creating low-cost 360-degree virtual reality videos for hospitals: a technical paper on the dos and don'ts. J Med Internet Res.

[37] Castanys TF, Carrión AJ, Gómez FR, García SC, Mascaray AM, Amela MJ, Bonet JB (2023). Effects of virtual tour on perioperative pediatric anxiety [IN PRESS]. Paediatr Anaesth.

[38] Eijlers R, Dierckx B, Staals LM (2019). Virtual reality exposure before elective day care surgery to reduce anxiety and pain in children: a randomised controlled trial. Eur J Anaesthesiol.

[39] Kruger P, Rosen D (2016). Parental presence at induction of anesthesia is feasible with minimal preparation and resources. Can J Anaesth.

[40] Capurso M, Ragni B (2016). Psycho-educational preparation of children for anaesthesia: a review of intervention methods. Patient Educ Couns.

[41] Astuto M, Rosano G, Rizzo G, Disma N, Raciti L, Sciuto O (2006). Preoperative parental information and parents' presence at induction of anaesthesia. Minerva Anestesiol.

[42] Connelly Y, Lotan R, Brzezinski Sinai Y (2022). Implementation of a personalized digital app for pediatric preanesthesia evaluation and education: ongoing usability analysis and dynamic improvement scheme. JMIR Form Res.

[43] Suarez-Lledo V, Alvarez-Galvez J (2021). Prevalence of health misinformation on social media: systematic review. J Med Internet Res.

[44] Stunden C, Stratton K, Zakani S, Jacob J (2021). Comparing a virtual reality-based simulation app (VR-MRI) with a standard preparatory manual and child life program for improving success and reducing anxiety during pediatric medical imaging: randomized clinical trial. J Med Internet Res.

[45] Smith V, Warty RR, Sursas JA (2020). The effectiveness of virtual reality in managing acute pain and anxiety for medical inpatients: systematic review. J Med Internet Res.

[46] Campbell-Yeo M, Dol J, McCulloch H (2023). the impact of parental presence restrictions on Canadian parents in the NICU during COVID-19: a national survey. J Fam Nurs.

[47] Foster JR, Lee LA, Seabrook JA (2022). Family presence in Canadian PICUs during the COVID-19 pandemic: a mixed-methods environmental scan of policy and practice. CMAJ Open.

[48] Yao J, Gong H, Zhao X, Peng Q, Zhao H, Yu S (2022). Parental presence and intranasal dexmedetomidine for the prevention of anxiety during anesthesia induction in children undergoing tonsillectomy and/or adenoidectomy surgery: a randomized controlled trial. Front Pharmacol.

[49] Jenkins BN, Fortier MA, Kaplan SH, Mayes LC, Kain ZN (2014). Development of a short version of the modified Yale Preoperative Anxiety Scale. Anesth Analg.

[50] Winterberg AV, Colella CL, Weber KA, Varughese AM (2018). The child induction behavioral assessment tool: a tool to facilitate the electronic documentation of behavioral responses to anesthesia inductions. J Perianesth Nurs.

[51] Matava CT, Gentry H, Simpao AF, Weintraub A (2023). Standardized Anesthesia InductioN Tool (SAINT) - the development and international adoption of an integrated electronic tool for documenting the induction of anesthesia in children [IN PRESS]. Paediatr Anaesth.

